# CSF GAP-43 as a biomarker of synaptic dysfunction is associated with tau pathology in Alzheimer’s disease

**DOI:** 10.1038/s41598-022-20324-2

**Published:** 2022-10-17

**Authors:** Qiang Qiang, Loren Skudder-Hill, Tomoko Toyota, Wenshi Wei, Hiroaki Adachi

**Affiliations:** 1 Department of Neurology, Cognitive Disorders Center, Huadong Hospital, Fudan University, Shanghai, China; 2 Yuquan Hospital, Tsinghua University School of Clinical Medicine, Beijing, China; 3 Department of Neurology, University of Occupational and Environmental Health School of Medicine, Kitakyushu, Japan

**Keywords:** Dementia, Alzheimer's disease

## Abstract

To test whether cerebrospinal fluid (CSF) growth-associated protein 43 (GAP-43) concentration is elevated in Alzheimer’s disease (AD) dementia and its associations with other hallmarks of AD, we examined the CSF GAP-43 measurements of 787 participants (245 cognitively normal (CN), 415 individuals with mild cognitive impairment (MCI) and 127 individuals with AD dementia) from the Alzheimer’s Disease Neuroimaging Initiative (ADNI) study. Associations were investigated between CSF GAP-43 and clinical diagnosis, Aβ/tau/neurodegeneration (AT(N)) status, CSF and blood biomarkers of AD, cognitive measurements and brain neuroimaging findings. CSF GAP-43 levels were increased in patients with AD dementia (mean, 6331.05 pg/ml) compared with the CN (mean, 5001.05 pg/ml) and MCI (mean, 5118.8 pg/ml) (*P* < 0.001) groups. CSF GAP-43 correlated with CSF phosphorylated tau 181(p-tau) (r = 0.768, *P* < 0.001), and had high diagnostic accuracy in differentiating tau positive status vs. tau negative status (area under the receiver operating characteristic curve, 0.8606). CSF GAP-43 was particularly elevated among individuals with tau positive status. High CSF GAP-43 was associated with longitudinal deterioration of cognitive scores and brain neuroimaging findings. CSF GAP-43 was associated with a clinical diagnosis of AD dementia and with an individual’s tau status, cognitive measurements and findings from neuroimaging. This study implies that CSF GAP-43 as a biomarker of synaptic dysfunction could predict the disease progression of AD patients.

## Introduction

Worldwide, Alzheimer’s Disease (AD) is one of the most common types of neurodegenerative disorders and is the leading cause of dementia. The neuropathological features of AD include extra cellular aggregation of amyloid β (Aβ) plaques, intracellular deposition of neurofibrillary tangles containing phosphorylated tau protein and synaptic loss^1^. Synapses are important for cognitive function, and synaptic impairment is one of the pathologic features of AD. A quantitative morphometric study has demonstrated a 25–35% decline of synaptic density in temporal and frontal cortical biopsies of patients (2–4 years) into the onset of AD^2^. In AD patients the synaptic loss primarily occurs in the neocortex and hippocampus^3^. Synaptic damage and cell loss leads to brain atrophy, and synaptic dysfunction is associated with cognitive impairment in AD^4^. Therefore, evaluating synaptic dysfunction in vivo would guide AD clinical research and could provide biomarkers for outcome assessment in AD clinical trials. In recent years, progress has been achieved to evaluate synaptic biomarkers in the cerebrospinal fluid (CSF). Depending on the localization of the synaptic protein, synaptic biomarkers can be divided into pre- and postsynaptic biomarkers. Several studies reported CSF concentrations of the presynaptic proteins, such as growth-associated protein 43 (GAP-43), synaptosomal-associated protein 25 (SNAP-25) and synaptotagmin-1, as well as postsynaptic protein neurogranin are altered in AD patients^5–8^. Among those synaptic proteins, GAP-43 plays an important role in the learning and memory storage process^9,10^.

In human, GAP-43 is a presynaptic protein consisting of 238 amino acids, and is located on the cytoplasmic side of the presynaptic membrane^11^. GAP-43 is phosphorylated by protein kinase C and then interacts with other proteins to facilitate axonal outgrowth and vesicular cycling^12^. In cultured hippocampus neurons, GAP-43 colocalizes with axonal marker tau protein^13^. GAP-43 is involved in axonal outgrowth, neuroplasticity and memory formation^10,14–16^. In the central nervous system, levels of GAP-43 protein are high in the cerebellum, neocortex, entorhinal cortex, hippocampus, olfactory bulb and retinal cells^17^. As a biomarker of synaptic dysfunction, previous studies reported that the concentration of CSF GAP-43 was elevated in AD patients^5,18–20^, and in this present study we analyzed whether CSF GAP-43 is associated with other CSF and blood biomarkers in AD and whether the baseline levels of CSF GAP-43 could predict changes in cognitive measurements and neuroimaging findings over time.

## Methods

### Study design

Data used in this study were downloaded from the Alzheimer’s disease Neuroimaging Initiative (ADNI) database (http://adni.loni.usc.edu/) in July 2021. ADNI is a public–private project launched in 2003 and led by principal investigator Michael W. Weiner, MD. The primary goal of the ADNI project is to develop clinical, neuroimaging, genetic and bio fluid biomarkers for the early diagnosis and monitoring of AD progression. Regional ethical committees of all participating institutions approved the ADNI study, and all methods were carried out in accordance with relevant guidelines and regulations. Written informed consent was obtained from all study participants.

### Participants

The inclusion and exclusion criteria for ADNI participants have been described previously^21^. Our study included 787 ADNI participants with available baseline CSF-GAP43 samples. The diagnostic criteria for cognitively normal (CN) controls, MCI and AD dementia were based on cognitive assessments. CN participants had a Mini-Mental State Examination (MMSE) score of 24 or higher and a Clinical Dementia Rating Scale (CDR) score of 0. Participants with MCI had an MMSE score of 24 or higher and CDR score of 0.5, objective memory loss was determined by delayed recall of the Wechsler Memory Scale Logical Memory 2, preserved activities of daily living, and absence of dementia. Participants with AD dementia had MMSE scores between 20 and 26 and CDR scores ranging from 0.5 to 1, fulfilling the National Institute of Neurological and Communicative Disorders and Stroke and the Alzheimer Disease and Related Disorders Association criteria for probable AD^22^.

### CSF GAP-43

CSF GAP-43 levels were measured by an in-house enzyme-linked immunosorbent assay (ELISA) method developed at the Clinical Neurochemistry Lab, University of Gothenburg, Sweden. ELISA was developed by using a combination of monoclonal GAP-43 antibody NM4 (coating antibody) and polyclonal GAP-43 antibody ABB-135 (detector antibody) to recognize the C-terminal of GAP-43. The ELISA assay range of CSF-GAP43 was from 312 to 20,000 pg/ml. Quality control CSF samples were from the Clinical Neurochemistry Laboratory, Sahlgrenska University Hospital, Mölndal, Sweden. Details of the ELISA assay of CSF GAP-43 has been described previously^5^.

### Biomarkers in CSF and plasma

CSF samples were collected by lumbar punctures from a subset of participants, and levels of CSF Aβ42, CSF total tau (t-tau), and CSF phosphorylated tau 181 (p-tau) were analyzed by electrochemiluminescence immunoassays (ECLIA) on a fully automated Elecsys cobas e 601 instrument as previously described^23,24^. CSF Aβ42, CSF p-tau and CSF t-tau were utilized to define amyloid pathology (A), tau pathology (T) and neurodegeneration (N) respectively according to the ATN framework^25^. The published cutoff values (CSF Aβ42 < 977 pg/mL, CSF p-tau > 27 pg/mL, CSF t-tau > 300 pg/mL)^26^ were used to define the positivity of A/T/N status.

Plasma Aβ42 and Aβ40 levels were measured at Bateman lab, Washington University School of Medicine. Immunoprecipitation of targeted Aβ isoforms was performed in the KingFisher (Thermo) automated immunoprecipitation platform by using an anti- Aβ mid-domain antibody (HJ5.1), and subsequently digested with Lys-N protease then analyzed by liquid chromatography tandem mass spectrometry (LC–MS/MS) as previously described^27^. Both plasma p-tau181 and neurofilament light (NfL) levels were analyzed by the Single Molecule array (Simoa) technique at the Clinical Neurochemistry Lab, University of Gothenburg, Sweden. Plasma p-tau181 was measured by an in-house assay, using a combination of two monoclonal antibodies (Tau12 and AT270) and targeted N-terminal to mid-domain forms of p-tau181^28^. Plasma NfL levels were analyzed by the Simoa technique using a combination of monoclonal antibodies, and purified bovine NfL was used as a calibrator for the measurements^29^.

### Neuroimaging

Structural brain imaging was performed by 3.0 T MRI scanners, T1 weighted images in NiFTI format were quantified by FreeSurfer version 5.1 for regional volumes according to the 2010 Desikan-Killany atlas^30^. Volumetric data of the medial temporal lobe, and hippocampus were used in our analysis and were adjusted for intracranial volume. Positron emission tomography (PET) with 18F-fluorodeoxyglucose (FDG) image data was used to identify hypometabolic regions related to pathological metabolic change in MCI and AD, and FDG-PET data was processed by Helen Wills Neuroscience Institute, University of California Berkeley and Lawrence Berkeley National Laboratory. The mean counts from composite metaROI (region of interest) of left angular gyrus, right angular gyrus, bilateral posterior cingulate, left inferior temporal gyrus, right inferior temporal gyrus relative to pons and cerebellar vermis reference region were adopted for FDG-PET assessment^31^.

### Cognition measurement

The cognition level of participants in the cohort was evaluated by the Mini-Mental State Examination (MMSE), the 11 item version of the Alzheimer Disease Assessment Scale Cognitive Subscale (ADAS-COG 11) and the CDR Scale Sum of Boxes (CDR-SB).

### Statistical analysis

Descriptive statistics between diagnostic groups were compared using ANOVA, Kruskal–Wallis, chi-square test according to the distribution of each variable. CSF GAP-43 data had a skewed distribution (based on visual inspection of the histogram) and were log transformed to produce a normally distributed dataset. The differences of log transformed CSF GAP-43 between tau negative and tau positive groups were examined by the student’s t test, differences between multiple groups were compared with one-way analysis of variance (ANOVA) and the Tukey post hoc test. Receiver operating characteristic (ROC) analyses were used to evaluate the diagnostic accuracy of tau status by CSF GAP-43, and DeLong test was performed to compare area under the curve (AUC) values. Marginsplot was created using Stata software to predict the probability of tau positive status based on CSF GAP-43 levels. Decision curve analysis was performed using the DCA function code in Stata software. Linear regression models were used to test associations between biofluid markers and CSF GAP-43. Linear mixed-effect models were used to test associations between CSF GAP-43 with longitudinal cognition scores, and imaging of brain structures. These models included the interaction between time and CSF GAP-43 as a predictor, and random slope and random intercept for time and unstructured covariance structure for random effects. All outcome variables in linear mixed-effect models were standardized to z scores so that the effects could be directly compared between associations. All regression analyses were adjusted for age, sex, years of education and APOE ε4 status, intracranial volume was also adjusted for in models involving imaging of brain structures. Two-sided *P* values < 0.05 were considered statistically significant, and Stata version 16 (College Station, TX) statistical software was used for all statistical analyses.

### Ethical approval

ADNI was approved by the regional ethical committees of all participating institutions (A complete listing of ADNI participating institutions can be found at: https://adni.loni.usc.edu/wp-content/uploads/how_to_apply/ADNI_Acknowledgement_List.pdf).

## Results

In this study we included 787 participants with available CSF GAP-43 measurements, the mean (SD) age was 72.35 (7.3) years old, and 416 (52.86%) were men. The mean (SD) education years of the participants was 16.27 (2.60) years, among them 430 (54.64%) were APOE ε4 allele negative (online supplemental Table 1). We further stratified the participants by clinical diagnosis, 245 participants were CN controls, 415 had MCI, and 127 had AD dementia, the demographic information, CSF biomarkers, neuroimaging findings and cognition scores of the participants are shown in Table 1. The baseline CSF GAP-43 levels differed significantly among the three clinical diagnostic groups (CN controls 5001.05 pg/mL; MCI 5118.80 pg/mL; dementia 6331.05 pg/mL; *P* < 0.001).

**Table 1:** Demographic data of the study population.

Variable	CN (n = 245)	MCI (n = 415)	AD (n = 127)	*P*-value
Age at baseline, years	72.91 (5.98)	71.37 (7.47)	74.48 (8.49)	< 0.001
Male sex, N (%)	111 (45.31%)	228 (54.94%)	77 (60.63%)	0.009
Education level, years	16.67 (2.48)	16.19 (2.63)	15.74 (2.67)	0.003
**APOE ε4 status, N** (**%)**				< 0.001
APOE ε4−/−, N (%)	174 (71.02%)	214 (51.57%)	42 (33.07%)	
APOE ε4+/−, N (%)	64 (26.12%)	156 (37.59%)	59 (46.46%)	
APOE ε4+/+, N (%)	7 (2.86%)	45 (10.84%)	26 (20.47%)	
**CSF biomarkers**				
Aβ42 level, pg/mL	1042.32 (378.39)	886.83 (336.40)	652.30 (260.62)	< 0.001
p-tau level, pg/mL	21.75 (9.42)	26.36 (14.38)	36.64 (16.18)	< 0.001
t-tau level, pg/mL	237.36 (92.57)	274.92 (128.71)	371.44 (154.61)	< 0.001
GAP-43 level, pg/mL	5001.05 (2706.21)	5118.80 (2826.12)	6331.05 (3126.55)	< 0.001
Brain neuroimaging*				
Hippocampus, mm^3^	7535.78 (873.39)	7063.45 (1111.48)	5924.88 (968.27)	< 0.001
Medial temporal lobe, mm^3^	20,741.68 (2504.99)	20,393.53 (2703.86)	17,770.57 (3148.32)	< 0.001
FDG-PET composite	1.32 (0.11)	1.26 (0.13)	1.06 (0.15)	< 0.001
**Cognitive score**				
MMSE	29.05 (1.17)	28.07 (1.71)	23.22 (2.03)	< 0.001
ADAS-Cog 11	5.69 (2.94)	9.22 (4.46)	20.74 (6.86)	< 0.001
CDR-SB	0.05 (0.15)	1.44 (0.87)	4.59 (1.70)	< 0.001

MCI, mild cognitive impairment; MMSE, Mini-Mental State Examination; ADAS-COG 11, Alzheimer Disease Assessment Scale–cognitive subscale; CDR-SB, CDR Scale Sum of Boxes; p-tau, phosphorylated tau 181; t-tau, total tau; FDG-PET, fluorodeoxyglucose-positron emission tomography. Data are presented as mean (SD) for continuous variables, and n (%) for categorical variables.

*MRI structural imaging measurements reported are unadjusted by total intracranial volume.

**Figure 1:**
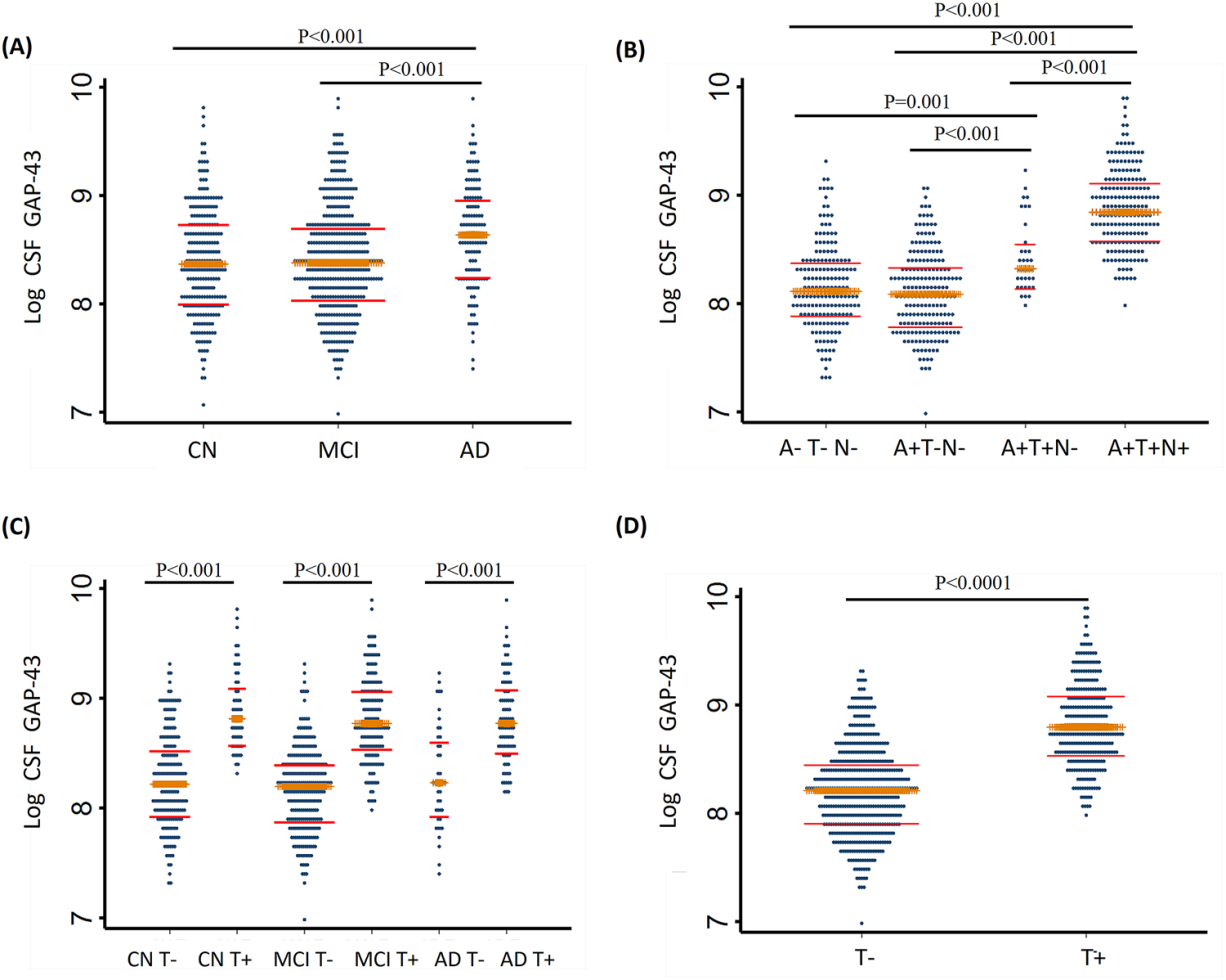
CSF GAP-43 according to clinical diagnosis and tau status. (**A**) Comparison of CSF GAP-43 between cognitively normal (CN), individuals with mild cognitive impairment (MCI) and Alzheimer’s disease (AD) dementia. (**B**) CSF GAP-43 in individuals according to Aβ/tau/neurodegeneration (ATN) profile. A represents amyloid pathology, T represents tau pathology, and N represents neurodegeneration, the cutoff values (CSF Aβ42 < 977 pg/mL, CSF p-tau > 27 pg/mL, CSF t-tau > 300 pg/mL) were used to define the positivity of A/T/N status respectively. (**C**) CSF GAP-43 in CN, and in individuals with MCI and AD Dementia, stratified by tau positive status (CSF p-tau > 27 pg/mL) (**D**) comparison of CSF GAP-43 between tau negative (T−) and tau positive (T+) status.

CSF GAP-43 levels were higher in the AD dementia group compared with the MCI and CN control groups, however there were no statistically significant differences between the MCI and CN control groups (Fig. 1A). We also compared CSF GAP-43 levels according to the participants’ CSF A/T/N profiles, CSF GAP-43 levels were higher in the A+T+N+ group compared to the A+T+N– group (*P* < 0.001), the A+T–N– group (*P* < 0.001) and the A–T–N– group (*P* < 0.001) (Fig. 1B). The A+T+N– group had higher CSF GAP-43 levels than the A+T–N– group (*P* < 0.001) and the A–T–N– group (*P* = 0.001), the difference between the A+T–N– group and the A–T–N– group was not statistically significant (Fig. 1B). Comparisons between groups according to the participant characteristics by clinical diagnosis and CSF tau status showed that CSF GAP-43 levels were higher in tau positive individuals with AD dementia than tau negative individuals with AD dementia (*P* < 0.001), tau positive individuals with MCI had higher CSF GAP-43 levels than tau negative individuals with MCI (*P* < 0.001), and CSF GAP-43 levels were higher in tau positive controls compared with tau negative controls (*P* < 0.001) (Fig. 1C). We further stratified the participants according to their CSF tau status, and tau positive participants had higher CSF GAP-43 concentrations than tau negative participants (*P* < 0.0001) (Fig. 1D).

**Figure 2:**
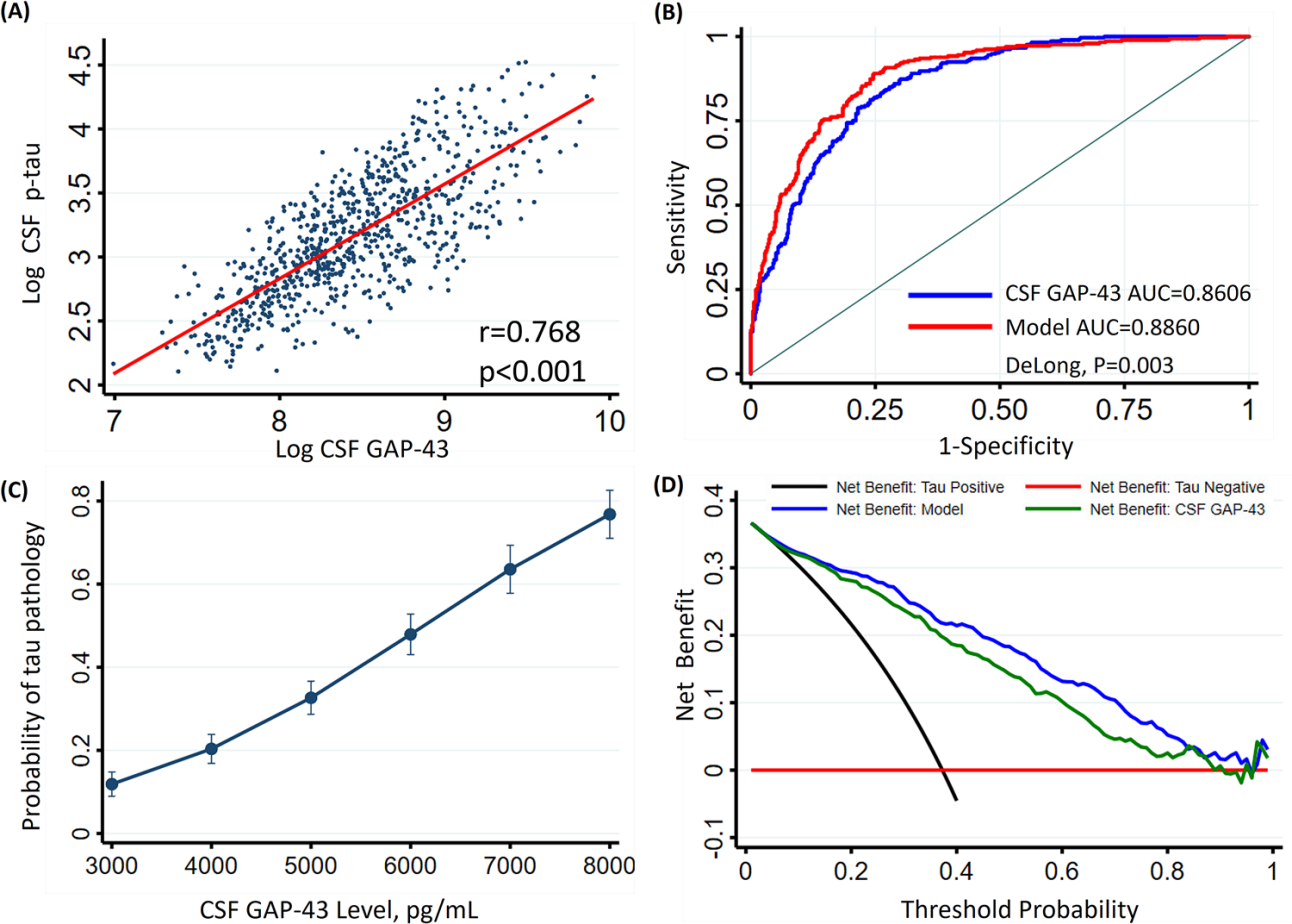
Association of CSF GAP-43 with tau pathology. (**A**) fit line is demonstrated in the cohort for the correlation between Log CSF GAP-43 and Log CSF p-tau. Pearson correlation was performed to acquire ρ and *P* values. (**B**) ROC analyses for discriminating tau pathology positive versus negative status were performed, and DeLong test was conducted to compare AUC values. Blue line: ROC curve of CSF-GAP43. Red line: ROC curve of a logistic regression model including CSF-GAP43, age, sex, years of education and APOE ε4 allele status. (**C**) probability of positive tau status predicted by CSF GAP-43 concentration. (**D**) decision curve for prediction of tau pathology. Red line: assumes no individuals have tau pathology. Black line: assumes all individuals have tau pathology. Green line: prediction model only includes CSF GAP-43. Blue line: prediction model includes CSF GAP-43, age, sex, education years and APOE ε4 genotype. Tau pathology positive was defined as CSF p-tau > 27 pg/mL. ROC, receiver operating characteristic; AUC, area under the curve.

CSF GAP-43 was correlated with high CSF p-tau (r = 0.768, *P* < 0.01) (Fig. 2A). We further stratified the participants by clinical diagnostic group, and CSF GAP-43 was correlated with high CSF p-tau in all diagnostic groups (online supplemental Fig. 1). CSF GAP-43 levels were also correlated with high CSF t-tau and high plasma p-tau levels. The strongest correlation of CSF GAP-43 was with CSF p-tau and CSF t-tau, however there was no correlation of CSF GAP-43 with CSF Aβ42, plasma Aβ42/40 ratio, or plasma NfL (Table 2). ROC analyses of tau positive cases versus tau negative cases demonstrated that the area under the curve (AUC) for CSF GAP-43 was 0.8606, in a logistic regression model adjusted for age, sex, years of education and APOE ε4 allele status the AUC improved to 0.886, which was statistically higher than that of CSF GAP-43 along (DeLong *P* = 0.003) (Fig. 2B). To estimate the predicted probability of tau positive status for the participants, we generated a plot based on logistic regression considering tau positive/negative status as an outcome and CSF GAP-43 levels as a predictor. It showed that CSF GAP-43 was a strong predictor for the probability of tau status (Fig. 2C). Decision curve analysis was performed for the binary outcome tau positive/negative, and it was seen clearly that the line corresponding to CSF GAP-43 had higher net benefit compared to assuming tau positive for all participants or tau negative for all participants. The line corresponding to the logistic regression model incorporated CSF GAP-43 with age, sex, years of education and APOE ε4 status, and had the highest net benefit across a wide range of threshold probabilities (Fig. 2D).

**Table 2:** Correlations between CSF GAP-43 and other biomarkers.

Baseline biomarkers	Baseline CSF GAP-43		
	β coefficient	*P* value	R^2^
CSF Aβ42	0.01	0.819	0.088
CSF p-tau	0.745	< 0.001	0.523
CSF t-tau	0.745	< 0.001	0.550
Plasma Aβ42/40	− 0.077	0.273	0.035
Plasma p-tau	0.086	0.015	0.068
Plasma NfL	0.04	0.355	0.063

Baseline levels of all biomarkers were standardized to z scores so that the effects could be directly compared. Data are β coefficients (with P values) from linear regression models which were used to test associations between CSF GAP-43 and other biomarkers, adjusted for age, sex, education level, and APOE ε4 genotype.

CSF, cerebrospinal fluid; p-tau, phosphorylated tau 181; t-tau, total tau; NfL, neurofilament light.

**Figure 3:**
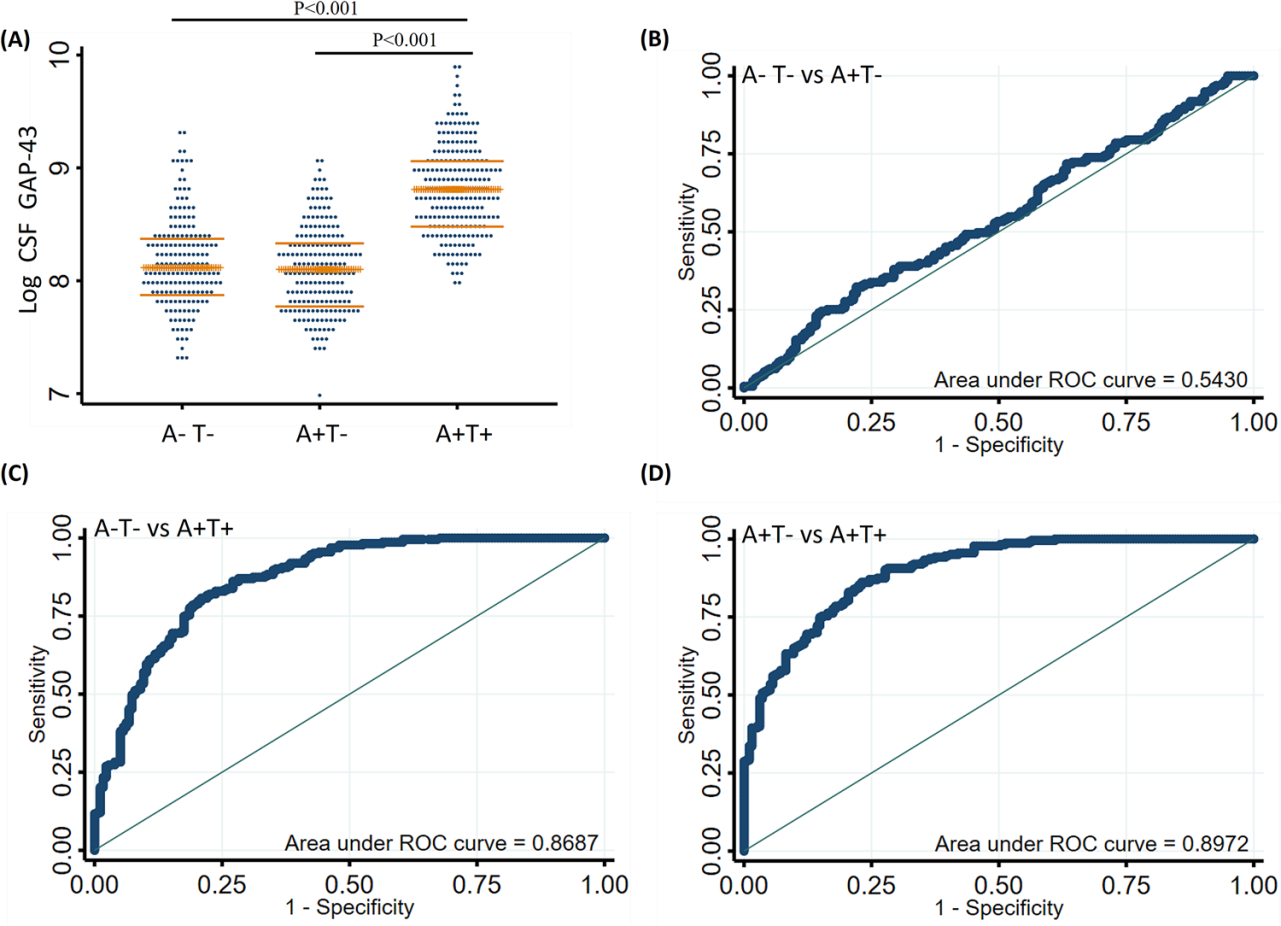
CSF GAP-43 according to amyloid and tau status. (**A**) comparison of CSF GAP-43 between individuals by Aβ/tau (AT) profile. (**B**) receiver operating characteristic (ROC) analyses were performed for CSF GAP-43 discriminating A–T– from A+T–. (**C**) ROC analyses were performed for CSF GAP-43 discriminating A–T– from A+T+. D, ROC analyses were performed for CSF GAP-43 discriminating A+T– from A+T+. A indicates amyloid pathology, T indicates tau pathology, and the cutoff values (CSF Aβ42 < 977 pg/mL, CSF p-tau > 27 pg/mL) were used to define the positivity of amyloid pathology and tau pathology respectively.

We further classified the participants based on the presence of amyloid pathology (A) and tau pathology (T) in CSF into three groups: A–T–, A+T–, A+T+. CSF GAP-43 levels were higher in the A+T+ group compared with the A–T– and A+T– group, there were no statistically significant differences of CSF GAP-43 levels between the A–T– and A+T-groups (Fig. 3A). We performed ROC analysis to assess the accuracy of CSF GAP-43 to discriminate between the different AT groups. CSF GAP-43 showed superior accuracy discriminating A–T– from A+T+ (AUC = 0.869, Fig. 3C) and A+T– from A+T+ (AUC = 0.897, Fig. 3D), compared with discriminating A–T– from A+T– (AUC = 0.543, Fig. 3B).

**Figure 4:**
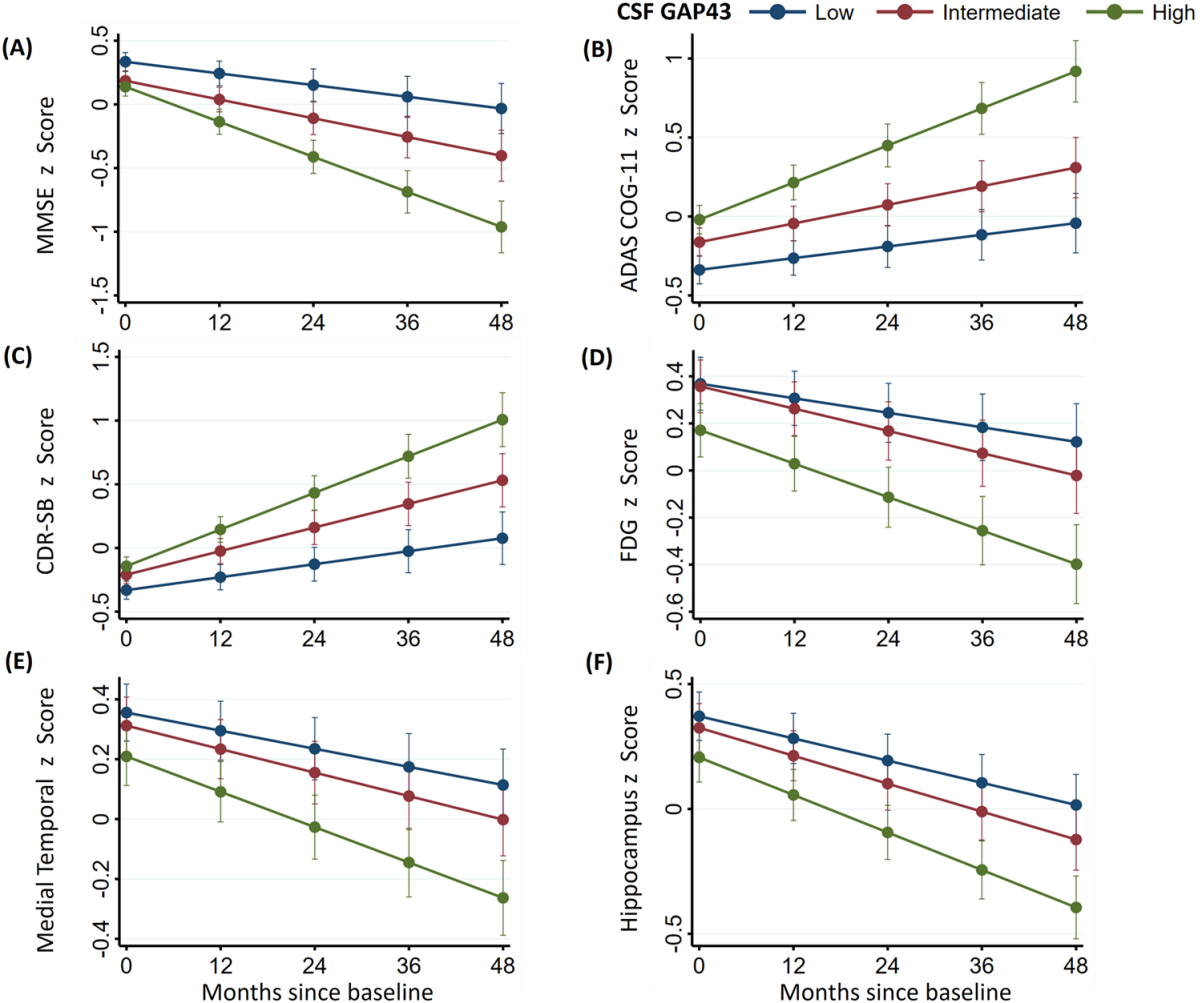
Trajectories of cognitive measurements and brain neuroimaging findings according to CSF GAP-43 tertile groups. CSF GAP-43 levels were divided into three tertiles: Low, Intermediate, High. Linear mixed-effects (LME) regression analyses were used to estimate the effects of CSF GAP-43 levels on cognitive measurements, brain imaging findings at baseline and over time. All LME analyses were adjusted for age, sex, education years and APOE ε4 genotype, as well as intracranial volume for the MRI structural imaging measurements. All outcome variables in liner mixed-effects models were standardized to z scores. Supplementary Tables 2–7 give details about baseline levels (intercept) and changes over time (slope) of those trajectories. MMSE represented Mini-Mental State Examination; ADAS-COG 11, Alzheimer Disease Assessment Scale–cognitive subscale; CDR-SB, CDR Scale Sum of Boxes; FDG, 18F-fluorodeoxyglucose.

**Figure 5:**
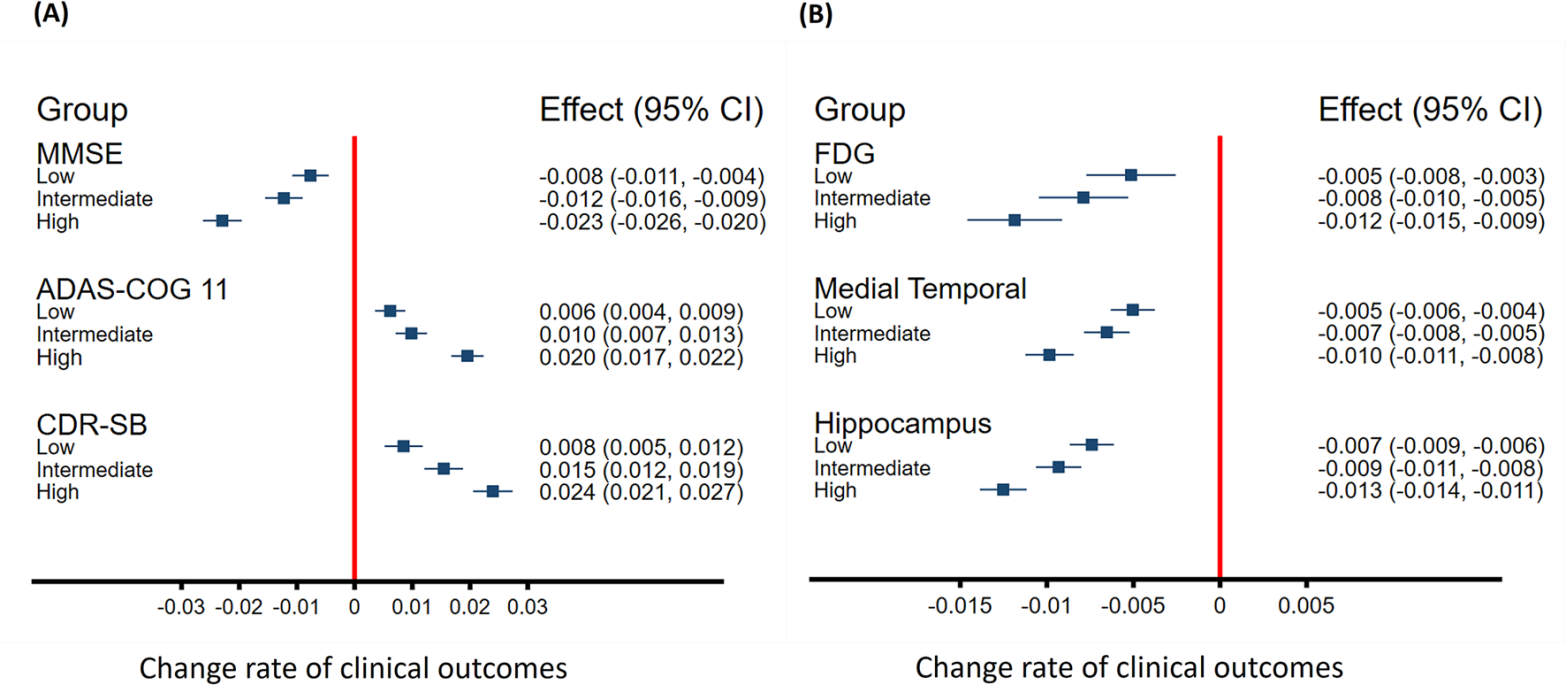
Estimated rates of changes in clinical outcomes among CSF GAP-43 tertile groups based on linear mixed-effects regression models. (**A**) monthly changes in MMSE, ADAS-COG 11, and CDR-SB scores, analyses were adjusted for age, sex, education years and APOE ε4 genotype. (**B**) monthly changes in FDG-PET composite ROIs, medial temporal lobe and hippocampus volumes, analyses were adjusted for age, sex, education years, APOE ε4 genotype, and intracranial volume for MRI imaging measurements. All outcome variables in linear mixed-effects models were standardized to z scores so that the effects could be directly compared. CSF GAP-43 levels were divided into three tertiles: Low, Intermediate, High. MMSE represented Mini-Mental State Examination; ADAS-COG 11, Alzheimer Disease Assessment Scale–cognitive subscale; CDR-SB, CDR Scale Sum of Boxes; FDG, 18F-fluorodeoxyglucose; ROI, region of interest.

Associations between CSF GAP-43 and longitudinal cognition, and neuroimaging findings are displayed in Fig. 4 (coefficients and P values of intercepts and slopes are reported in online supplemental Tables 2–7). At baseline, high CSF GAP-43 levels were associated with reductions in MMSE scores, increases in ADAS-COG11 and CDR-SB scores, decreases in FDG-PET composite ROIs, and smaller hippocampus and medial temporal lobe volume. Over time, high CSF GAP-43 levels were associated with accelerated reduction in MMSE, increase in CDR-SB and ADAS-Cog scores, reduction in hippocampus and medial temporal volume, and greater decline of FDG-PET composite ROIs. CSF GAP-43 levels were divided into three tertiles: low level, intermediate level and high level, estimated rates of change in cognition scores and neuroimaging across the three groups (Low, Intermediate and High) were demonstrated in Fig. 5A,B. Comparisons between groups demonstrated that high CSF GAP-43 groups had faster deterioration rates than low CSF GAP-43 groups in terms of both cognition scores and neuroimaging findings.

## Discussion

The present study showed that CSF GAP-43 was elevated in the clinical AD dementia group and in tau positive individuals. CSF GAP-43 was correlated with CSF p-tau, CSF t-tau and plasma p-tau. CSF GAP-43 levels were much higher in the tau positive group compared to the tau negative group, which distinguished tau positive status from tau negative status. The 2018 AD research framework proposed a biomarker classification system called the Aβ/tau/neurodegeneration (AT(N)) system, which defined AD as an AD continuum based on core neuropathological changes in vivo^25^. If only an Aβ abnormality was present (A+T–N–) this would put the individual at a place on the AD continuum called AD pathological change. If both Aβ and tau abnormalities were present, but neurodegeneration was absent (A+T+N–), the individuals could be considered as having AD even without exhibiting signs of neurodegeneration. If all three domains of Aβ, tau and neurodegeneration were present (A+T+N+), this would reflect a more advanced stage on the AD continuum compared to an A+T+N– profile. In our study, we demonstrated that CSF GAP-43 levels increased along the AD continuum. Besides, CSF GAP-43 levels were also associated with cognitive deficits and neuroimaging finds both at baseline and during longitudinal follow up.

There is much evidence that synaptic loss is correlated with cognitive decline in AD, and synaptic dysfunction is one of the earliest detectable changes in many neurodegenerative diseases, which may appear even before neuronal loss^32^. The significant role of synaptic dysfunction in the pathology of AD promotes the analysis and quantification of synaptic proteins. GAP-43 is a synaptic membrane protein which plays an important role in the regulation of synaptic plasticity, learning and memory functionality^16^. Previous studies reported that the concentration of CSF GAP-43 was increased in AD^5,18–20^, and our results are in line with these reports. Our study demonstrated that CSF GAP-43 was correlated with CSF p-tau, CSF t-tau and plasma p-tau, which might reflect a common pathogenic process between GAP-43 and tau pathology. Our study also demonstrated that CSF GAP-43 could efficiently discriminate tau pathology status. A+T– represented a stage of amyloid pathological change and A+T+ indicated an advanced stage with both amyloid and tau pathologies present. CSF GAP-43 was particularly pronounced in A+T+ compared to A+T– individuals, thus CSF GAP-43 could efficiently discriminate between A+T– and A+T+ stages. It has been suggested that high concentrations of CSF t-tau represent axonal degeneration and high concentrations CSF p-tau represent the increased formation of neurofibrillary tangles, and that these two events are associated^33,34^. As CSF GAP-43 was highly correlated with CSF p-tau and CSF t-tau, this may indicate that increased CSF GAP-43 concentration is associated with the degeneration of axons or presynaptic terminals, or the regeneration of axons and/or synapses^35^.

According to the amyloid cascade hypothesis, the soluble oligomer Aβ initiates tau pathology, and tau pathology leads to neuronal dysfunction and cell loss^36^. A previous study utilized synaptosomes from the cortex of postmortem human subjects and transgenic rat models of AD to elucidate the time sequence of Aβ and tau pathology in synaptic terminals. This study demonstrated that Aβ accumulated in synaptic terminals in the early stages of AD, and these changes appeared before the accumulation of synaptic p-tau, the accumulation of p-tau in synaptic terminals occurred in the late stages of AD^37^. Aβ initiated synaptic dysfunction via tau pathology, and without the presence of tau pathology there would not be resulting synaptic dysfunction and memory impairment^38^. In our study we showed that CSF GAP-43 were correlated with CSF p-tau and plasma p-tau, and there was no correlation between CSF GAP-43 and CSF Aβ42. This could be partially explained by CSF GAP-43 being a biomarker of synaptic dysfunction, and the accumulation of tau pathology being correlated with synaptic dysfunction and memory impairment. The accumulation of Aβ42 initiated all of these events.

Aβ pathology and tau pathology are two major pathological hallmarks of AD, and tau aggregation is the primary pathological feature of a category of clinically heterogeneous neurodegenerative disorders termed tauopathies^39^. Tauopathies include but are not limited to AD, progressive supranuclear palsy (PSP), corticobasal syndrome (CBS) and some types of frontotemporal lobar degeneration (FTD), and pathogenic tau aggregation has different characteristics among these disorders^40^. A study of 662 individuals found that CSF GAP-43 concentration was increased significantly in clinical AD and CBS patients, while there were no concentration differences between both the control and clinical PSP groups, and behavioral variant FTD (bvFTD), and amyotrophic lateral sclerosis (ALS) with FTD groups^5^. The differences in the structural characteristics of tau aggregation and cellular localization among those disordered might lead to heterogeneous concentrations of CSF GAP-43 in comparison to the control group.

In this study we also found that CSF GAP-43 was associated with several cognitive and neuroimaging hallmarks of AD at baseline and over time. Specifically, high CSF GAP-43 levels were associated with MMSE, ADAS-COG 11, and CDR-SB scores at baseline as well as with accelerated deterioration of those cognitive measures longitudinally. Synapses are important for cognitive function, and synaptic loss is one of the pathologic features of AD^2^. Synaptic dysfunction has been associated with cognitive impairment in AD^4^. Our study showed that on neuroimaging, high CSF GAP-43 levels were associated with smaller hippocampus and medial temporal lobe volume, lower FDG-PET values at baseline, and accelerated reduction in hippocampus and medial temporal volume, greater decline of FDG-PET values over time. A neuroimaging study reported that synaptic density in the hippocampus area was decreased in AD patients^41^. Synaptic activity could be measured by cerebral glucose metabolism, and FDG-PET measures low cerebral glucose metabolism in regions of interest in AD patients, which associated with concurrent cognitive decline^31^.

The present study is limited by lacking data from other neurodegenerative disorders, which prevents our ability to test the disease specificity of CSF GAP-43 for AD, and whether CSF GAP-43 is associated with other neuropathological biomarkers such as α-synuclein, TDP-43. Another limitation of this study is its lack of evaluation of other tauopathies such as FTD, PSP, and CBS, which limits analysis of association between CSF GAP-43 and tau pathology in other tauopathies. For tau pathology assessment, in this study due to lack of tau PET finings, we used p-tau in CSF to quantify tau pathology, which was associated with CSF GAP-43 levels. However, previous studies have demonstrated that findings of tau PET neuroimaging were more closely associated with neurodegeneration than CSF tau^42^. Our study also lacks analysis of the longitudinal changes of CSF GAP-43 levels in AD, and longitudinal changes of CSF GAP-43 levels would better depict the features of CSF GAP-43 in AD. Finally, in this study due to lack of other synaptic biomarker’s measurements data (such as CSF SNAP-25, CSF neurogranin), we only analyzed CSF GAP-43’s association with other core AD fluid biomarkers.

In conclusion, the CSF concentration of synaptic membrane protein GAP-43 was specifically elevated in tau positive individuals and could be used a biomarker for synaptic dysfunction. In addition to Aβ/tau/neurodegeneration (AT(N)) neuropathological biomarkers, CSF GAP-43 could be included as another neuropathological measurement of synaptic dysfunction in AD. In clinical study scenarios of AD, CSF GAP-43 could be used as another outcome measurement to predict longitudinal disease progression.

## Supplementary Information

Supplementary Information.

## Data Availability

Data used in the current study were originally obtained from the online repository of Alzheimer’s Disease Neuroimaging Initiative (ADNI) (http://adni.loni.usc.edu/). The data generated during processing and analyzing are available from the corresponding author on reasonable request.

## Code availability

All codes used during data analysis in this study are available from the authors upon request.
