# Supplementary material for: CSF GAP-43 as a biomarker of synaptic dysfunction is associated with tau pathology in Alzheimer’s disease
Source: Sci Rep. 2022 Oct 17;12:17392. doi: 10.1038/s41598-022-20324-2 (PMC9576773; doi:10.1038/s41598-022-20324-2)
Supplement: Supplementary file 1 — Supplementary Information. [file 41598_2022_20324_MOESM1_ESM.docx]

**Supplementary Table 1. Demographic data of the study participants (n = 787)**

| **Variable** | **Total (n=787)** |
| --- | --- |
| Age at baseline, years | 72.35 (7.30) |
| Male sex, N (%) | 416 (52.86%) |
| Educational level, years | 16.27 (2.60) |
| APOE ε4 status, N (%) |  |
| APOE ε4-/-, N (%) | 430 (54.64%) |
| APOE ε4+/-, N (%) | 279 (35.45%) |
| APOE ε4+/+, N (%) | 78 (9.91%) |
| CSF biomarkers |  |
| Aβ42 level, pg/mL | 883.41 (359.41) |
| p-tau level, pg/mL | 26.59 (14.22) |
| t-tau level, pg/mL | 278.80 (130.95) |
| GAP-43 level, pg/mL | 5277.77 (2874.69) |
| Brain neuroimaging ^*^ |  |
| Hippocampus, mm^3^ | 7031.78 (1145.57) |
| Medial temporal lobe, mm^3^ | 20142.59 (2871.25) |
| FDG-PET composite | 1.25 (0.15) |
| Cognitive score |  |
| MMSE | 27.59 (2.55) |
| ADAS-Cog 11 | 9.97 (6.73) |
| CDR-SB | 1.52 (1.75) |

Abbreviations: MMSE, Mini-Mental State Examination; ADAS-COG 11, Alzheimer Disease Assessment Scale–cognitive subscale; CDR-SB, CDR Scale Sum of Boxes; CSF, cerebrospinal fluid; p-tau, phosphorylated tau 181; t-tau, total tau; FDG-PET, fluorodeoxyglucose-positron emission tomography. Data are presented as mean (SD) for continuous variables, and n (%) for categorical variables.^*^ *Measurements reported from MRI structural imaging are unadjusted by total intracranial volume.*

**Supplementary Table 2. Baseline levels and longitudinal changes of MMSE z Score according to CSF GAP-43 tertile group**

|  | **Low** | **Intermediate** | **High** |
| --- | --- | --- | --- |
| **Intercept,β** | 0.335 | **0.185** | **0.139** |
| **Intercept, p-value** | NA | **0.004** | **<0.001** |
| **Slope,β** | -0.008 | **-0.012** | **-0.023** |
| **Slope, p-value** | NA | **0.046** | **<0.001** |

Baseline levels (intercept) and over time changes (slope) of MMSE z Score by CSF GAP-43 tertile group. Comparisons with reference group were derived from linear mixed effects models, adjusted for age, sex, education level, and APOE ε4 genotype. MMSE outcome measures were standardized to z scores, CSF GAP-43 levels were divided into three tertiles: Low, Intermediate, High, and Low was the reference group for comparison (P values < 0.05 were considered statistically significant and highlighted in bold). MMSE indicates Mini-Mental State Examination.

**Supplementary Table 3. Baseline levels and longitudinal changes of ADAS-COG 11 z Score according to CSF GAP-43 tertile group**

|  | **Low** | **Intermediate** | **High** |
| --- | --- | --- | --- |
| **Intercept,β** | -0.338 | **-0.162** | **-0.020** |
| **Intercept, p-value** | NA | **0.006** | **<0.001** |
| **Slope,β** | 0.006 | 0.010 | **0.020** |
| **Slope, p-value** | NA | 0.057 | **<0.001** |

Baseline levels (intercept) and over time changes (slope) of ADAS-COG 11 z Score by CSF GAP-43 tertile group. Comparisons with reference group were derived from linear mixed effects models, adjusted for age, sex, education level, and APOE ε4 genotype. ADAS-COG 11 outcome measures were standardized to z scores, CSF GAP-43 levels were divided into three tertiles: Low, Intermediate, High, and Low was the reference group for comparison (P value s< 0.05 were considered statistically significant and highlighted in bold). ADAS-COG 11 indicates Alzheimer Disease Assessment Scale–cognitive subscale.

**Supplementary Table 4. Baseline levels and longitudinal changes of CDR-SB z Score according to CSF GAP-43 tertile group**

|  | **Low** | **Intermediate** | **High** |
| --- | --- | --- | --- |
| **Intercept,β** | -0.331 | **-0.210** | **-0.143** |
| **Intercept, p-value** | NA | **0.018** | **<0.001** |
| **Slope,β** | 0.008 | **0.015** | **0.024** |
| **Slope, p-value** | NA | **0.004** | **<0.001** |

Baseline levels (intercept) and over time changes (slope) of CDR-SB z Score by CSF GAP-43 tertile group. Comparisons with reference group were derived from linear mixed effects models, adjusted for age, sex, education level, and APOE ε4 genotype. CDR-SB outcome measures were standardized to z scores, CSF GAP-43 levels were divided into three tertiles: Low, Intermediate, High, and Low was the reference group for comparison (P values < 0.05 were considered statistically significant and highlighted in bold). CDR-SB indicates CDR Scale Sum of Boxes.

**Supplementary Table 5. Baseline levels and longitudinal changes of FDG z Score according to CSF GAP-43 tertile group**

|  | **Low** | **Intermediate** | **High** |
| --- | --- | --- | --- |
| **Intercept,β** | 0.368 | 0.357 | **0.171** |
| **Intercept, p-value** | NA | 0.890 | **0.017** |
| **Slope,β** | -0.005 | -0.008 | **-0.012** |
| **Slope, p-value** | NA | 0.140 | **<0.001** |

Baseline levels (intercept) and over time changes (slope) of FDG-PET composite ROIs z Score by CSF GAP-43 tertile group. Comparisons with reference group were derived from linear mixed effects models, adjusted for age, sex, education level, and APOE ε4 genotype. FDG-PET composite ROIs measures were standardized to z scores, CSF GAP-43 levels were divided into three tertiles: Low, Intermediate, High, and Low was the reference group for comparison (P values < 0.05 were considered statistically significant and highlighted in bold). FDG indicates 18F-fluorodeoxyglucose; ROI, region of interest.

**Supplementary Table 6. Baseline levels and longitudinal changes of medial temporal volume z Score according to CSF GAP-43 tertile group**

|  | **Low** | **Intermediate** | **High** |
| --- | --- | --- | --- |
| **Intercept,β** | 0.356 | 0.312 | **0.209** |
| **Intercept, p-value** | NA | 0.527 | **0.037** |
| **Slope,β** | -0.005 | -0.007 | **-0.010** |
| **Slope, p-value** | NA | 0.108 | **<0.001** |

Baseline levels (intercept) and over time changes (slope) of medial temporal volume z Score by CSF GAP-43 tertile group. Comparisons with reference group were derived from linear mixed effects models, adjusted for age, sex, education level, and APOE ε4 genotype and intracranial volume. Medial temporal volume outcome measures were standardized to z scores, CSF GAP-43 levels were divided into three tertiles: Low, Intermediate, High, and Low was the reference group for comparison (P values < 0.05 were considered statistically significant and highlighted in bold).

**Supplementary Table 7.Baseline levels and longitudinal changes of hippocampus volume z Score according to CSF GAP-43 tertile group**

|  | **Low** | **Intermediate** | **High** |
| --- | --- | --- | --- |
| **Intercept,β** | 0.371 | 0.325 | **0.206** |
| **Intercept, p-value** | NA | 0.508 | **0.020** |
| **Slope,β** | -0.007 | **-0.009** | **-0.013** |
| **Slope, p-value** | NA | **0.040** | **<0.001** |

Baseline levels (intercept) and over time changes (slope) of hippocampus volume z Score by CSF GAP-43 tertile group. Comparisons with reference group were derived from linear mixed effects models, adjusted for age, sex, education level, and APOE ε4 genotype and intracranial volume. Hippocampus volume outcome measures were standardized to z scores, CSF GAP-43 levels were divided into three tertiles: Low, Intermediate, High, and Low was the reference group for comparison (P values < 0.05 were considered statistically significant and highlighted in bold).

**Supplementary Figure 1. Correlations between CSF GAP-43 and CSF p-tau by clinical diagnosis**


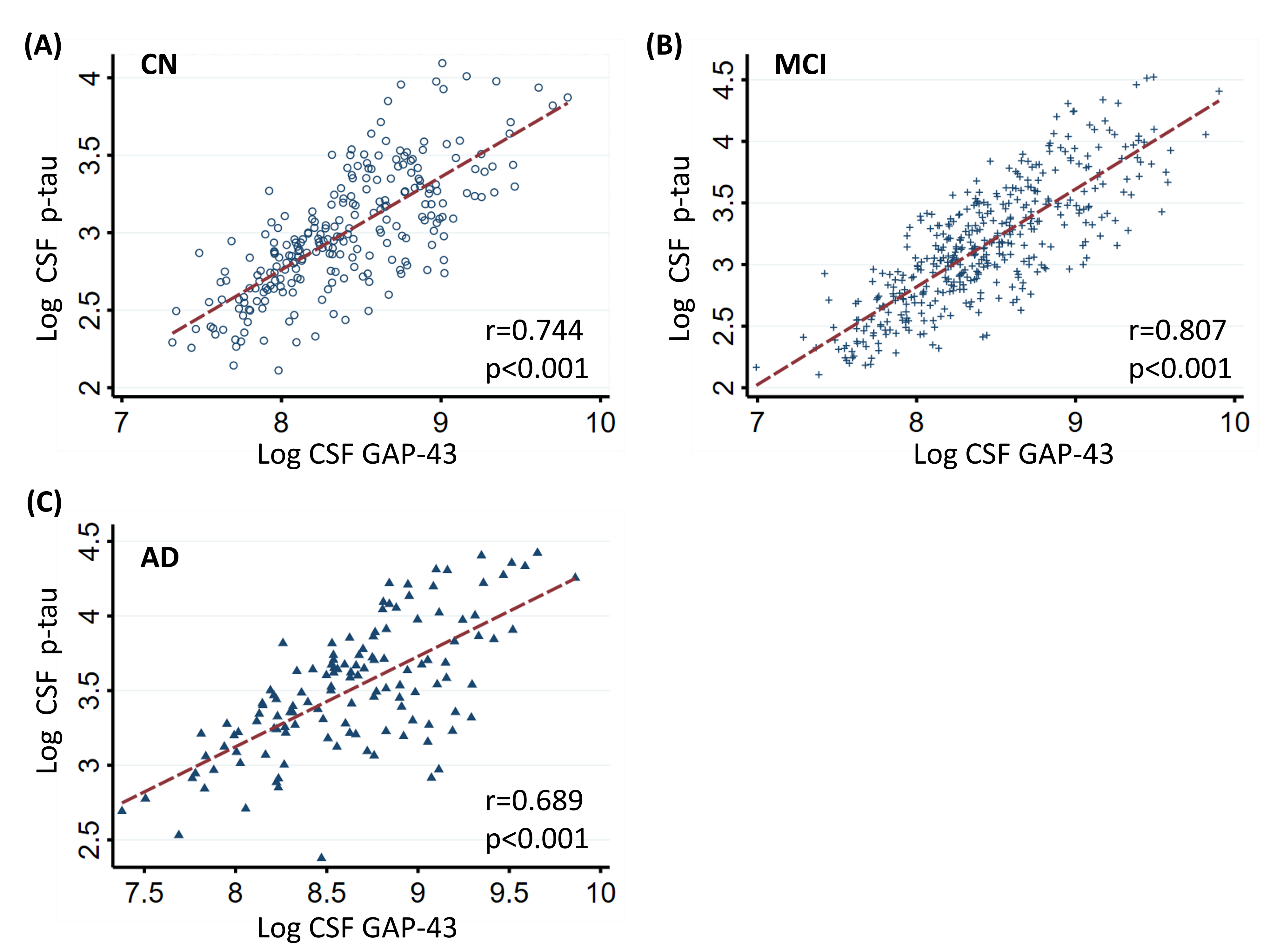


Fit lines are demonstrated in the cohort for the correlations between Log CSF GAP-43 and Log CSF p-tau for individual diagnostic groups. Pearson correlation was performed to acquire ρ and P values. A, correlation between CSF GAP-43 and CSF p-tau in the cognitively normal (CN) group. B, correlation between CSF GAP-43 and CSF p-tau in the mild cognitive impairment (MCI) group. C, correlation between CSF GAP-43 and CSF p-tau in the Alzheimer’s disease (AD) group.
